# 

**Published:** 1880-04

**Authors:** 


SURG-GENLS-OFFS

Vol. XVI. April, 1880. No. 1.

### THE BISTOURY.

Thad S. Up de Graff, M.D.
EDITOR.

ELMIRA, N.Y.

